# What Is It Like to Be a Bass? Red Herrings, Fish Pain and the Study of Animal Sentience

**DOI:** 10.3389/fvets.2022.788289

**Published:** 2022-04-27

**Authors:** G. J. Mason, J. M. Lavery

**Affiliations:** Integrative Biology, University of Guelph, Guelph, ON, Canada

**Keywords:** fish, sentience, consciousness, pain, welfare

## Abstract

Debates around fishes' ability to feel pain concern *sentience*: do reactions to tissue damage indicate evaluative consciousness (conscious affect), or mere nociception? Thanks to Braithwaite's research leadership, and concerns that current practices could compromise welfare in countless fish, this issue's importance is beyond dispute. However, nociceptors are merely necessary, not sufficient, for true pain, and many measures held to indicate sentience have the same problem. The question of whether fish feel pain – or indeed anything at all – therefore stimulates sometimes polarized debate. Here, we try to bridge the divide. After reviewing key consciousness concepts, we identify “red herring” measures that should not be used to infer sentience because also present in non-sentient organisms, notably those lacking nervous systems, like plants and protozoa (P); spines disconnected from brains (S); decerebrate mammals and birds (D); and humans in unaware states (U). These “S.P.U.D. subjects” can show approach/withdrawal; react with apparent emotion; change their reactivity with food deprivation or analgesia; discriminate between stimuli; display Pavlovian learning, including some forms of trace conditioning; and even learn simple instrumental responses. Consequently, none of these responses are good indicators of sentience. Potentially more valid are aspects of working memory, operant conditioning, the self-report of state, and forms of higher order cognition. We suggest new experiments on humans to test these hypotheses, as well as modifications to tests for “mental time travel” and self-awareness (e.g., mirror self-recognition) that could allow these to now probe sentience (since currently they reflect perceptual rather than evaluative, affective aspects of consciousness). Because “bullet-proof” neurological and behavioral indicators of sentience are thus still lacking, agnosticism about fish sentience remains widespread. To end, we address how to balance such doubts with welfare protection, discussing concerns raised by key skeptics in this debate. Overall, we celebrate the rigorous evidential standards required by those unconvinced that fish are sentient; laud the compassion and ethical rigor shown by those advocating for welfare protections; and seek to show how precautionary principles still support protecting fish from physical harm.

## Introduction

Debates around fishes' ability to feel pain are essentially debates about consciousness. In other words, the central issue is: when fish react to actual or threatened tissue damage, does this indicate true pain, with its “phenomenal” character [“an unpleasant sensory and emotional experience”, e.g., (1)]? Or just mere nociception: an unconscious process by which noxious stimuli are responded to? [c.f. e.g., (2); see also (3–7)]. Today, thanks in part to the trailblazing work of Dr. Victoria Braithwaite celebrated in this Special Topic collection, there is no disputing what an important issue this is, and also no argument as to whether bony fishes possess functioning nociceptors [e.g., rainbow trout (*Oncorhynchus mykiss)*: (8, 9), goldfish (*Carassius auratus)*: (10), common carp (*Cyprinius carpio*): (11)]. But the question of whether fish are *aware* of noxious stimuli, and feel true pain, remains contested and controversial, stimulating considerable debate. Views can be polarized: at one extreme some argue that fish have no awareness of anything at all, including pain (Key in prep., pers. comm), while at the other extreme some argue that they feel not only pain but also fear (12) and even maybe joy (13). Yet *most* seem uncertain: 83% of the 43 responses to Key (14) in *Animal Sentience*, for example, do not take a firm stance on whether or not fish can feel pain. And this reflects a much broader, harder problem: that the functions of consciousness are still not understood. Trying to identify what would make for stronger evidence of sentience was therefore one of Victoria's last pieces of scholarly work (15).

So, as authors of yet another “fish sentience” review, what can we add that will constructively promote her legacy? Our aim is to celebrate the rigorous evidential standards required by those remaining unconvinced that fish are sentient [e.g., (6, 16)], and to leverage the high levels of evidence they require into a research agenda for the future. But we also laud the compassion and ethical rigor shown by those advocating welfare protections for fish [e.g., (17–19)], and seek to show how the high current levels of agnosticism about fish sentience are consistent with adopting practical guidelines that aim to protect fish [e.g., see discussions from (16, 20–23)]. To put this in the context of our own views, personally and professionally we treat fish as though sentient (e.g., we are working on a zebrafish enrichment and welfare project in which Victoria was involved). But we do this because we are taking a precautionary approach; like her we feel that fish should be treated as if sentient [e.g., (24)]–despite not yet being convinced there is strong evidence that fish *definitely are sentient*. This paper therefore aims to illustrate why being uncertain, while simultaneously treating fish as sentient, is a reasonable stance; and to outline what types of data could decrease this uncertainty in the future. In this way we hope to honor our friend and colleague.

To do this, first we review some key consciousness concepts, including “sentience” (a term with two meanings); and the deductive “theory heavy” vs. inductive “theory neutral” approaches typically used to infer capacities for pain in non-human animals. We follow this by identifying types of measure that should not be used to infer sentience, because they can be performed by organisms reasonably assumed to be non-sentient (such as spines disconnected from brains, decerebrate mammals and birds, and humans in states of unawareness). We argue that these are “red herrings”: measures that add little to debates about fish pain or animal sentience. We then suggest some experimental approaches more likely to rely on sentience, and thus to be valid indicators: certain operant tasks, tasks reliant on working memory, methods that ask animals to self-report their feelings, and higher order abilities like episodic memory. We also suggest how future research could test the validity of these potential indicators. Finally, returning to the fish pain debate, we attempt to alleviate concerns raised by key skeptics that sentience is irrelevant for welfare consideration, and that classifying fish as sentient will be harmful to people. We also summarize what the indicators most likely to be valid reveal about fish.

### What Is It Like to Be a Bass? Some Consciousness Basics

Primary or “phenomenal” consciousness (often abbreviated to P-consciousness) is raw experience or sensation: the ability to feel or be aware, sometimes described – to parallel Nagel's famous essay “What is it like to be a bat?” (1974) – as the “what it is like” aspect of a state [e.g., (25), p. 32–37]. Many equate this with “sentience”, using this term to mean all forms of P-consciousness [e.g., (26–30)].

When consciousness can influence actions (including, for humans, speech), it is often termed “access consciousness” [e.g., (26)]. It is therefore *through* access consciousness (e.g., self-report in humans) that researchers make inferences about P-consciousness. Combined with its subjective, private nature, this means that P-consciousness is most readily (indeed perhaps only) empirically detectable in humans, via experiments that rely on self-report [e.g., (25, 31), p. 187–210; (32), p. 224, and many others]. An important sub-type of access consciousness comprises higher order forms [cf. e.g., (29)], involving self-reflection or introspection: self-consciousness (an awareness of self), for example, or the “mental time travel” implicated in the episodic recall of past experiences [e.g., (33, 34)]. Such higher order forms of consciousness are generally seen as reliant on P-consciousness [e.g., the taste of past food, the visual recall of past foraging sites, the sensory inputs from one's own body, etc.; e.g., (35)]. Passing experimental tests for self-reflection or self-awareness is therefore usually taken as evidence of P-consciousness – although what *types* of P-consciousness are necessary to pass such tests depend on what exactly is being tested for (something we discuss further below).

**Figure 1:**
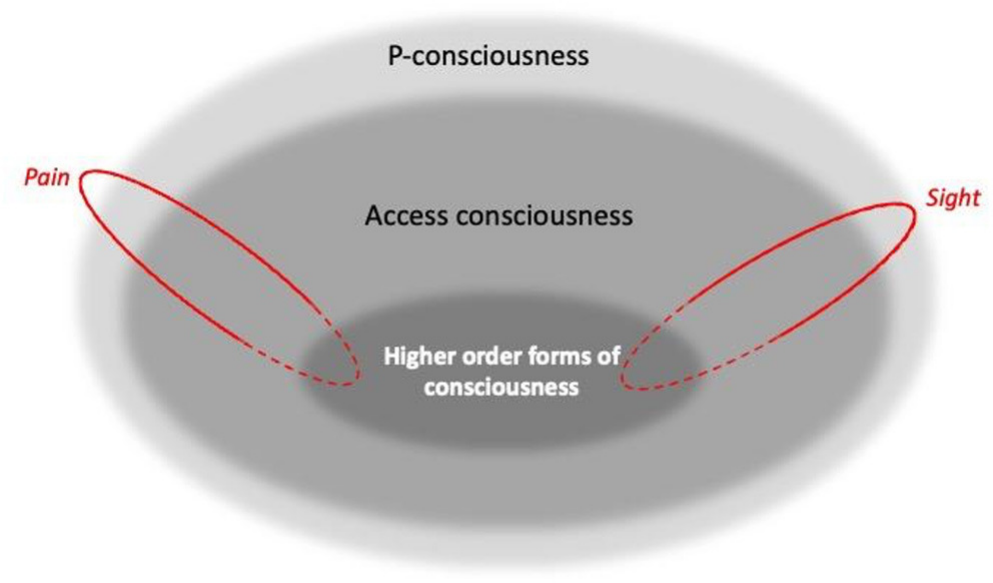
A Venn diagram illustrating three broad, arguably hierarchical levels of consciousness [Inspired by Blackmore and Troscianko (26), Block (25), Birch (33), Birch et al. (36), Ginsburg and Jablonka (37)]. Boundaries between levels are blurred to capture how they likely vary by degrees rather than being “all or none”. Spanning the hierarchical levels are components that represent independent, dissociable sensory modalities and affective states, such as sight and pain, shown here in red (along with many others: see text). For these, solid outlines indicate parallel processing, while dashed outlines indicate the potential for diverse dimensions to be bound together (as for example during multi-modal recognition).

Putting these concepts together, in this paper we therefore assume hierarchical levels of consciousness as illustrated in Figure 1's Venn diagram. Here, higher order forms of self-reflection (“I know that I feel pain”), as well as non-introspective self-reported states (“Pain is present”), reveal and rely on P-consciousness. However, P-consciousness may occur without higher order self-reflection, or indeed perhaps without any form of access consciousness. This conception follows e.g., Blackmore and Troscianko (25), Birch (33), Birch et al. (36), and Ginsburg and Jablonka (37). But it is at odds with some other authors: as for so many other aspects of consciousness, there is no consensus here, and so some argue there can be no P-consciousness without higher order forms [e.g., (38–40)]; no P-consciousness without access consciousness [e.g., (41)]; and even that there can be access consciousness without P-consciousness [e.g., (42, 43)]. Nevertheless, this figure provides a model of our practical assumptions (and those often used by researchers interested in animal consciousness), including that self-report provides a window into P-consciousness, and that failing tests for higher-order consciousness (or even other forms of access consciousness) does not prove a lack of sentience.

Figure 1 also illustrates another feature of consciousness: we drew the boundaries between hierarchical levels as blurred because they seem to exist in degrees rather than being “all or none” [cf. e.g., (29)]. For example, we know from human research that some types of stimuli may be on the threshold of conscious detectability, with subjects being barely aware of them [e.g., (44)]. Furthermore, P-consciousness can be a reversible state (as well as a trait) that we lose during anesthesia or sleep; and researchers studying its waxing and waning in humans find that this state is graded: not just present or absent, but instead occurring by degrees [e.g., (25); p. 115, 119, 189; (45), p. 33; (37), p. 187]. By analogy, this could suggest that the *trait* of consciousness can also vary by degrees.

In addition, as well as having hierarchical levels, consciousness also comprises parallel, non-hierarchical components such as the phenomenal pain and phenomenal vision (sight) shown in Figure 1. Although often bound or unified together to give a multi-modal representation of the world [e.g., (46)], these are independent and dissociable. Thus, in their heuristically valuable framework, Birch et al. (36) proposes that “perceptual richness” (the perception of aspects of the environment), and “evaluative” or affective richness (emotional experiences with valence, such as pain), are categories or dimensions of P-consciousness. In turn, each of these broad dimensions can be broken down into components, with sight being dissociable from conscious olfactory or proprioceptive experience, pain being distinct from hunger or pleasure, and so on. Other authors make similar distinctions [see e.g., (45), p. 54; (37), p. 7, 203], and data from humans clearly illustrate the separability of these components. For example, people who lack sight because of retinal or visual cortex damage can still consciously experience sound, smell, touch, pleasure, pain and so on [e.g., (32)]; people who lack phenomenal olfaction (perhaps because of olfactory cortex damage) can still taste, detect olfactory irritants that cause pain, and experience all other forms of P-consciousness [e.g., (47)]; people with certain cortical or thalamic lesions may become unable to perceive tactile, thermal, or high pressure somatosensory stimuli to the body [e.g., (48)], yet despite this numbness and lack of proprioception, still have all other forms of P-consciousness; and finally, people who lack the capacity to feel pain (due to a lack of receptors or central change in how pain signals are processed) likewise are still able to feel pleasure, and to consciously see, hear, taste, and so on [e.g., (48–50)].

Appreciating these different components is important in several ways for the fish pain debate. First, it illustrates how the word “sentience” is used to refer to two different concepts: a potential source of confusion. As mentioned above, some use this term to mean all forms of P-consciousness. But others (including ourselves) use sentience to refer to *just* the sub-type that is most ethically relevant: the dimension that Birch et al. term “evaluative”, or the capacity for felt emotions [e.g., (51–54)]. Second, because P-consciousness comes in these diverse dissociable forms, this means that evidence for one component tells us little (and perhaps nothing at all) about the presence of other components. Thus, evidence for visual awareness tells us nothing about the presence of olfactory awareness, for instance, or the ability to feel pain. This is not just an abstract issue. Appreciating such components is useful because it highlights how the cognitive data typically treated as evidence for P-consciousness in animals (sentience in its broadest sense), reveal little or nothing about *affective*, evaluative consciousness (sentience in its narrower, more ethically relevant sense). For example, mirror self-recognition tests probably indicate some forms of P-consciousness: those reliant on sight and phenomenal proprioception. But this does not reveal anything at all about an animal's capacity for pain or the other forms of conscious affect at the heart of sentience. In the section What *Could* Be Evidence of P-Consciousness and, More Narrowly, Sentience? we develop this argument further, and use it to propose some new types of experiment.

## Inferring Sentience In Non-Humans

For non-human animals, for whom we cannot use verbal self-report, researchers interested in inferring sentience typically take one of two approaches. One is to look for what are argued to be neurological prerequisites for sentience (i.e., particular structures or types of organization within the brain). This is a deductive approach that assumes that we know what these prerequisites are (thanks to research on adult humans), and is what Birch (33) would term “theory heavy”. A second approach–one especially useful for species lacking clear homology with humans – is to look for behavioral and cognitive responses that are at least consistent with sentience, and use these to make inferences: an inductive approach that would be called “theory neutral” by Birch (33). In the fish pain debate, the former approach is most often used by those arguing that fish cannot feel pain (or indeed anything at all), while the latter is most often used by those arguing that they do. In both cases, however, the protagonists are limited by their underlying assumptions.

Thus some authors have argued that fish do not have the types of brain necessary to be capable of pain, and these follow the first approach. Early versions based their argument on fishes' lack of our mammalian six-layered cortex and the assumption that this is crucial for any type of P-consciousness (3, 4, 6, 7, 14, 55). This stance thus equally rules out the possibility of any kind of P-consciousness in, for example, birds [which do not have a layered cortex at all, despite forebrain cyto-architecture of arguably similar complexity (56)]; in reptiles (which do have a layered cortex, but one with “only” three layers [e.g., (57)]); and of course all invertebrates (no matter how neurologically or behaviorally complex). This in turn raises an obvious question: do we know *for sure* that having six cortical layers represents the crucial, unique requirement for consciousness? And this in turn flags a general problem with such approaches: even if we can say that a neurological system with property X is sufficient for consciousness, this tells us nothing about the capacity of a system with 95% of that property, or 90%, or 80%, and so on [after (5, 33)].

Key and Brown (58) propose instead an agenda of “identifying the algorithm (sequence of neural functions) necessary for subjective experience and then seeking to define the specific neural structures (e.g., neural architectures and neural circuitry) that could possibly execute that algorithm among different species”. This seems sensible – if, and only if, it can escape from the problem outlined above. But even then, it is an extremely challenging task. For one, because humans are currently the best (only?) model in which P-consciousness can be studied, this approach is limited to principles derived from understanding human brains. Any neural correlates of P-consciousness must also disentangle it from the self-report behaviors used to access it experimentally [e.g., (25), p. 87; (45), p. 45]. Additionally, human consciousness researchers need to agree on what does or does not constitute good evidence for different hypotheses, and then systematically test them: a process that is still very much ongoing [e.g., (37), p. 142–147; (59, 60)]. Until then, there is no consensus on what neurological substrates are required [e.g., (45), p. 21; (37), p. 142–146], and so to quote Blackmore and Troscianko [(25), p. 260], “we should not just guess which features are needed for consciousness”.

Perhaps more fruitful is to look for behavioral or cognitive responses that seem consistent with sentience. This is the approach typically taken by those arguing that fish do feel pain. For example, Sneddon et al. (61) developed a list of behavioral, and also physiological, responses (e.g., rubbing, limping, or guarding; self-administration of analgesia; paying a cost to avoid a stimulus; etc.) that they argue would demonstrate pain perception in mammals and so should be assumed to do so in fish. However, this “theory neutral”, inductive approach is also problematic. As Key and Brown (62) cogently summed up: “the difficulty here of course is distinguishing whether the behavior truly demonstrates an underlying experience of pain”. Similar concerns are raised by Birch (33). And so as LeDoux and Brown (39) summarize, “deciding whether a non-verbal behavior reflects conscious vs. unconscious cognitive processes requires not only that the behavior be explainable in terms of conscious processes, *but also that non-conscious explanations are inadequate*” (our emphasis). This issue is important because, as we review in the section *Red Herrings:* Responses That Do Not Require Sentience, a large corpus of research shows that many superficially persuasive behavioral phenomena can actually occur without P-consciousness. Identifying responses that indicate sentience thus involves looking for types of affective response that humans can *only* make when they are aware [cf. e.g., (36, 39, 63, 64) and others]. The search for these is still on-going (see sections *Red Herrings:* Responses That Do Not Require Sentience and Discussion and Conclusions: Applying Our Approach to the Fish Pain Debate). But a second, complementary strategy is important too: identifying responses that do *not* require P-consciousness, so that these can be ruled out as likely “red herrings”. This is the focus of our next section.

## *Red Herrings:* Responses That Do Not Require Sentience

For a response to be used as evidence of pain, we need to know that it could not just reflect mere nociception. This understanding has already led most of those interested in this topic away from measuring physiological responses to harm [long recognized as automatic reactions that do not require awareness; e.g., (65)], and instead toward behavioral or cognitive measures whose nature and functionality is more likely to rely on sentience. But even here, are researchers being stringent enough? To assess this, we must identify responses that do not require sentience. And to do this, we need data from subjects assumed not to have sentience, or indeed any kind of P-consciousness.

**Figure 2:**
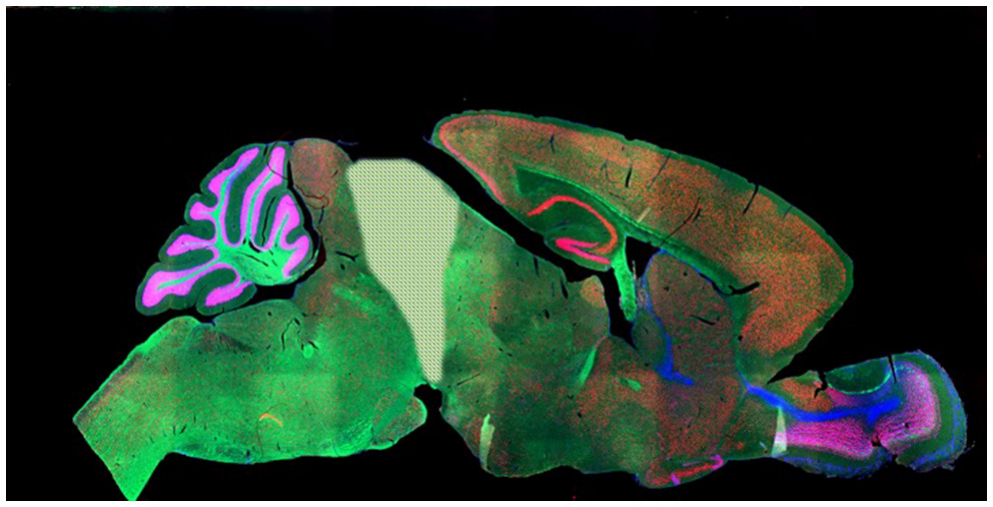
After Woolf (71), showing what is removed in a decerebrate rodent: everything to the right of the pale shaded area (thus cerebral cortices, subcortical gray matter, hippocampus, olfactory bulbs and diencephalon). The pale shaded area (superior colliculi) may be removed too. Note that despite its superficial complexity, the mammalian cerebellum is not involved in P-consciousness [(45), p. 54–55, 83]. Such subjects are generally argued to be pain-free and thus not to need anesthesia for further surgeries [e.g., (5, 72)]. Note that the same is not true for decorticate mammals subject to less severe surgery (e.g., losing only the cortical layers of the cerebra but retaining the thalamus): anesthesia is required for these. (Note that while we find data from these subjects very revealing, we also acknowledge that this work–along with that on spinally transected rats– is highly invasive and disturbing). Photo: Mouse brain, NIH National Institute of Child Health and Human Development.

For these, here we look to four types of subject: plants and protozoa (P), spines (S), decerebrate mammals (D) and unaware humans (U), a group we came to term “S.P.U.D. subjects”. To explain these choices, let us lay out the underlying assumptions. First we are assuming that P-consciousness requires a nervous system, such that responses by organisms without one (e.g., plants) are occurring without consciousness. This is not a consensus view [e.g., (66)], but it is the conventional one [cf. e.g., (37), p. 192; (67)]. Second, in animals with brains, we assume that responses that can be performed by the peripheral nervous system alone (e.g., the spine) do not require P-consciousness. This is not an assumption that organisms must evolve brains to be sentient [though many hold this to be reasonable, e.g., (45), p. 153–161; (68)], but an assumption that if organisms do have brains, *then* these brains are required for any sentience or broader P-consciousness [cf. e.g., (33, 65, 69)]. It is also based on evidence that damage to the peripheral nervous system does not impair P-consciousness in humans. Third, in mammals and birds, we assume that P-consciousness requires their cerebra [e.g., (59, 70)], such that any responses made by decerebrate subjects (Figure 2) cannot require sentience. Again, this is not an assumption that organisms must evolve cerebra to be sentient, but instead an assumption that for mammals and birds which have evolved to have these, they are essential for any sentience or broader P-consciousness. Though not all agree [e.g., (73)], this view is sufficiently widely-held, and with enough certainty, that it already informs research guidelines [e.g., (5, 72); see Figure 2]. Fourth and finally, we take responses performed by human subjects who are unaware of stimuli, for instance because they are anesthetized, asleep, or being exposed to subliminal cues they report as undetectable, as occurring without P-consciousness [cf. e.g., (69) and many others]. Again, this is an assumption, and its reliance on self-report is not perfect. The presence of residual, low levels of awareness is sometimes raised as a concern in such studies [e.g., (32), p. 21, 201–204; (37), p. 220; (74, 75)], as is distinguishing the “not experienced” from the “experienced but forgotten” [e.g., (37), p. 134]. Furthermore, our use of such data assumes that the absence of the *state* of P-consciousness (in us, a conscious, sentient species) reveals responses that do not require the *trait* of P-consciousness – and perhaps this is incorrect [c.f. (76)]. Nevertheless, such experiments are often used to study the nature of P-consciousness in humans; they use diverse manipulations to modify awareness (suggesting findings are not artifacts of a single methodology); humans' verbal reports provide unique insights into their subjective experiences; and furthermore, using human consciousness research to yield measures for use in animals is an orthodox approach [e.g., (63)]. In addition, the evidence below does not rely on these human data alone.

The types of behavioral response performed by such S.P.U.D. subjects are compelling (and sometimes unsettling). That they are able to show unlearned avoidance or approach responses is perhaps the least surprising [and consequently, that reflex withdrawal can occur independent of the experience of pain has long been appreciated; e.g., (5, 54)]. Thus, plant stems and leaves will move away from adverse cues like shade, and toward light (77). Similar responses occur in single-celled organisms [e.g., (78)], such as “backwards jerks” if *Paramecia* encounter AC shock (79). The reflex retraction of limbs from noxious stimuli has long been known to occur in spinally-transected cats, rats and humans, despite no involvement of these subjects' brains [e.g., (80), reviewed in (5)]; and they occur in decerebrate rats too [e.g., (71)]. When fed, decerebrate chicks “followed the grain with striking pecking precision when it was moved in front of them by a tweezer” (81). Likewise, “blindsighted” humans, unable to see because of damage to the visual cortex, are still able to avoid walking or reaching into obstacles, as well as to visually track or grasp stimuli that they report that they cannot see [(32), p. 33, 90; although as outlined in the next section, the position of such obstacles cannot be remembered]. Further unconditioned reactions to harmful stimuli in decerebrate mammals are notable because of their seemingly affective nature. Decerebrate animals can react to noxious stimuli “by flight or attack” (82). Decerebrate rats, “respond to noxious stimuli with a flexion withdrawal response, vocalization, turning to the site of the injury, licking or biting the site of the injury, complex escape response and attack responses”, although removing the noxious stimulus causes immediate return to passivity or grooming as if nothing had occurred [(71); see also (83) for similar reports]. They show startle responses to sudden sounds [e.g., (84)]. And decerebrate chicks “emitted contentment calls [sic] when warm and distress calls [sic] when cold” (81).

Such unlearned responses to stimuli can also be *modulated* in S.P.U.D. subjects, including by emotionally-relevant cues: they are not fixed and stereotyped. For example, faced with a startling stimulus, “jump” reflexes, and increases in heart rate and skin conductance (reflecting the sympathetic activation of sweat glands) are typically greater in fearful than relaxed humans, including subjects exposed to distressing images. Yet such images can still have this modulatory impact on the startle reflex even when presented in a way that precludes their conscious perception [e.g., (85–87)]. Thirsty humans also drink more (and rate the drink as more positive) if exposed to happy faces than angry faces, even when these are subliminal (88). Likewise, decerebrate rats show a greater ingestive response to sucrose if food deprived rather than sated (89). Furthermore, tail withdrawal reflexes in decerebrate and spinally transected rats are reduced by morphine (83, 90). And even in plants like the sensitive mimosa, whose leaves close when touched, responses to aversive stimuli like lit matches are dampened when the leaves are sprayed with lidocaine (91). These responses by S.P.U.D subjects thus show that modulation of avoidance or ingestive behaviors by affectively-relevant manipulations does not require sentience.

Being able to discriminate between presented stimuli does not seem to require P-consciousness either. For example, the same mimosa plants mentioned above habituate to repeated stimulation, but this appears to be stimulus-specific: thus after a plant habituates to repeated water drops, ceasing to react to them, their leaves will still close in response to a new stimulus-finger touch (92, 93). Tendrils of the perennial vine *Cayratia japonica* are also more likely to coil around neighboring non-self plants than neighboring self plants, again revealing discrimination (94). Decerebrate chicks show the same preferences for moving over still objects, and for particular colors, as intact birds (81). Humans exposed to cues that they have no awareness of can also still discriminate between them, if asked to choose: they report feeling as if they are completely guessing, yet they respond correctly significantly above chance. Thus, subjects can do this with masked, subliminal and other visual cues presented to prevent them being consciously perceived [(25), p. 188–192]. Humans with blindsight can also correctly identify, at levels above chance, whether two items are the same or different, which of two items is the larger, direction of movement, whether something is protruding or receding in a presented figure, the nature of an item portrayed in an image, and even the emotional expressions of faces that they cannot consciously perceive (but which they subconsciously imitate) [(32), p. 6–21]. Subjects with no conscious sense of smell due to olfactory bulb damage (“blindsmell”) can also discriminate and identify odors, despite no phenomenal olfaction (47); and similarly subjects with “numbsense” (no somatosensory awareness) can make relevant discriminations: e.g., correctly identifying the bodily location of an applied stimulus they cannot consciously feel [e.g., (95) citing (48, 96)]. Thus discriminating between available stimuli does not require P-consciousness. (If stimuli are not *presently* available though, but instead were presented a short interval before the task, such abilities seem lost: something expanded on in the section What *Could* Be Evidence of P-Consciousness and, More Narrowly, Sentience?).

Discrimination during some *learned* tasks can also occur without awareness, as we outline next. Thus, simple Pavlovian conditioning, in which subjects associate a predictive cue (a “CS”) with a reinforcer (a “UCS”), generally does not require P-consciousness. Thus, this type of learning may occur in plants, for instance: pea seedlings can learn to grow toward a breeze that predicts light, or away from one that predicts no light [(97); although this has not been replicated: (98)]. Likewise, *Paramecia* exposed to vibration-shock pairings will learn to jerk backwards to just the vibration alone (79), while a related protozoan can learn to avoid bright locations paired with a shock (99). And *Paramecia* can show conditioned approach to a needle previously baited with food [(100); see also (101)]. The pairing of a cue with a shock can also be learned by mammalian spinal columns [e.g., (102)]; by cats lacking mid- and forebrains (103, 104); by other decerebrate mammals [e.g., rabbits: (105), guinea pigs: (106)]; and by humans who are asleep [e.g., (107)]. Decerebrate cats are even able to show discrimination: they could learn (albeit slowly) that one tone predicted a shock while another did not, and when this contingency was reversed, they could learn the reversal (103). Human subjects exposed to “backward masked” visual cues and similar manipulations that prevent awareness, along with people with blindsight, can all similarly learn to associate a shock with visual cues that they report they cannot see [e.g., (108–111)]. And finally, in a reward-motivated form of Pavlovian learning, subjects asked to rapidly categorize words (e.g., male vs. female names) came to respond more accurately if the words of each category were reliably preceded by a consistent visual CS (e.g., one CS before all female names, another CS before all male) – even when these CSs were subliminal (112). Perhaps unsurprisingly then, diverse forms of Pavlovian conditioning have also been shown in mammals anesthetized with drugs that induce unconsciousness in humans [e.g., (113–115), and reviewed (116)].

One sub-type of Pavlovian learning has often been suggested to differ: trace conditioning. Here, there is no temporal overlap between CS and UCS: the CS (including its offset) fully precedes the UCS. It had been long believed that human subjects need to explicitly understand the relationship between CS and UCS to show this type of learning [see (74, 117)]: an understanding requiring them to be aware of both stimuli. However, some of the studies in the preceding paragraph involved no overlap between CS and UCS, despite authors not always using the term “trace conditioning” here [e.g., see (108–110) as examples; possibly also 101]. Learning to avoid certain flavors presented as CSs can also occur even if the UCS of sickness is induced after an interval, in animals rendered unconscious with anesthesia [e.g., (118)]. Furthermore, trace conditioning can occur in decerebrate guinea pigs [e.g., (119)], and humans who are asleep (120). Human data also indicate that trace conditioning can occur with subliminal CSs (112, 121). As a final example modeled on human blindsight, in macaques whose visual cortices were experimentally damaged so that parts of their visual fields were blind, trace conditioning of a visual cue to a juice reward occurred even if that cue was presented to the animals' blind fields (122). Nevertheless, specific sub-types of trace conditioning involving learning the length of the delay, perhaps thanks to working memory, may still require awareness: see Birch (33) and next section.

What about learning that an *action* leads to a reinforcer? Following Grau and colleagues, here we distinguish what they term “instrumental” learning, where innate responses to UCSs become modified in form and timing (123), from true “operant” learning in which the subject acquires arbitrary responses not in their unlearned, evolved behavioral repertoires (such that a diverse range of responses can potentially be reinforced *de novo* by valenced stimuli, and correspondingly, a variety of difference reinforcers can be used to train a particular response). Neither form of learning occurs in plants (98), but instrumental learning of this type does occur in spines. Spinally transected rodents can learn to retract their hindlegs for particular periods of time, to avoid shocks to the foot (123, 124). These subjects also show “positive transfer”: if they have already learned to do this with one leg, they are faster to do it with the other (123). Furthermore, if the hindlimb of a spinally transected cat encounters an obstacle as it is swung forward, then the spinal cord rapidly learns to flex the leg to a greater extent to reduce contact with the obstacle (125). Similar instrumental learning occurs in decerebrate animals. Thus, decerebrate ferrets can learn to alter how they flex a limb to avoid colliding with an obstacle [reviewed (126)]. Another case involves modifying compensatory “up-hill responses”. If placed on a slope, with their heads lower than their rumps, rats typically alter their position via a compensatory “up-hill response”. Normal rats readily learn to suppress this response if it is punished with an electrical shock to the tail, but so too do decerebrate rats (82). The spontaneous, stereotyped pecking of pigeons can also become directed to a stimulus key by reinforcement, even in decerebrated birds (127). Furthermore, such birds can learn to modify that response according to a light signaling whether or not this pecking will yield food (a discriminative stimulus, DS) (128). Finally, in another study of monkeys whose visual cortices were experimentally damaged to induce blindness in parts of their visual field, Kato et al. (129) tested whether instrumental learning could occur. They successfully used a conditioned (secondary) visual stimulus (paired with juice) presented in the lesion-affected blind field, to reinforce looking in a particular direction.

As for true operant learning involving more arbitrary responses, this seems far more equivocal. It seems uninvestigated in plants or spines [with (123) stating: “there is no evidence that spinal mechanisms can meet the criteria of operant learning. …. In vertebrates, such learning may require a brain”]. Likewise, operant learning is rarely studied in decerebrate animals – in all three cases, perhaps because of the challenges of shaping responses in organisms that do not spontaneously emit variable behavior. There are just two rare potential exceptions here. Research on decerebrate duck embryos found they were able to learn to flex their feet in order to avoid shocks to their wings (130), although whether this is a true operant vs. an instrumentally modified reflex is not clear. In contrast decerebrate rats could not learn to climb onto a platform to escape from a tank of water [while decorticate rats, who retained the thalamus and other structures (Figure 2) could; (131)]. The remainder of our evidence here comes from unaware humans. In one complex study, human subjects could manually alter the temperature part of their hand was exposed to, and were told to make this temperature as stable as possible. However, in reality they were being reinforced for something different (e.g., increasingly greater tolerance of a hotter and hotter temperature), the reinforcement being a potentially painful increase. Subjects learned this task – despite not even realizing it was a task – but independently of their ability to report the reinforcer (31). The authors argued that awareness of reinforcing stimuli may therefore not be necessary for operant learning. In another experiment, subjects were set an operant button-pushing task, with backward masking used to render visual discriminative stimuli (DS+ and DS- image cues) non-visible. Subjects could still learn to push the button during only the DS+ condition (121, 132), thus learning to respond when shown subliminal cues associated with monetary rewards, and to withhold responding when shown subliminal cues associated with monetary penalties. However, whether these subjects could have learned to push this button *de novo*, without any instruction and [in (121)] without explicit feedback on their financial losses and gains, is unknown [and indeed seems unlikely: (132)]. Furthermore, not everyone has been able to replicate such results [c.f. (133, 134)].

Other related studies have assessed the effects of unconscious rewards on the *performance* rather than acquisition of operant tasks; thus tasks that were instructed rather than acquired by reinforcement. Some were simple motor responses, others more cognitively demanding and reliant on working memory. In one, former opioid addicts were found to lever-press to receive very small doses of morphine they reported they could not feel (135). Another used a manual gripping task, in which subjects were told that forceful grasps would win them bigger rewards, but that the sums at stake would vary. Presented with subliminal images of the monetary rewards on offer, subjects responded more forcefully when these were large rather than small [(136); see also (137)]. Ziauddeen et al. (138) obtained similar findings using subliminal cues of high/low value food items. In these four studies, more intense responding thus occurred for high than low rewards, despite subjects being unaware of subliminal incentive signals. However, it is unknown whether such differential operant responding would occur if subjects had not been instructed how and why to perform that task but instead had to learn it *de novo* via associative conditioning. Again using subliminal images of high/low financial rewards, Zedelius et al. (139) also found that high rewards promoted better performance of a working memory (word memorization) task, Likewise, subjects were better at an executive task reliant on working memory (140), and at switching between different tasks (141), if the potential rewards were high, even when they were unaware of the signaling images. But once more, subjects were given instructions on what the task involved, as well as explicit feedback on how well they were doing financially. Finally, Correa et al. (142) found that subjects could learn which of two buttons to press, based on images of money rewards that were designed to be subliminal (thanks to backward masking). However, again whether they could have learned to press button at all without instructions (i.e., with only subliminal rewards) is less clear; and importantly, the backward masking seemed unsuccessful in this study, as there was evidence of residual awareness. Overall, this leaves the ability of purely subliminal rewards to modify actions (with no explicit feedback) unclear. The ability of subliminal incentives to *condition* tasks this complex, from scratch, via associative learning also seems unknown.

Overall, a range of learned and unlearned behavioral responses thus do not seem to require P-consciousness, and even responses that “appear” emotional do not require sentience. Of course, *absolute certainty* about the absence of sentience in the plants and protozoa, spines, unaware humans and decerebrate mammals reviewed here, can always be challenged. Sentience is a subjective, private state that currently is not directly measurable: to be as definitive about its absence is nearly as hard as to be definitive about its presence. (And this makes the responses of decerebrate mammals especially disturbing, because so often exposed to harms that would cause pain in intact subjects). Nevertheless, these caveats acknowledged, it is highly defensible to propose that any responses performed by S.P.U.D. subjects do not require sentience, especially for responses that occur across the whole diverse group. And as a consequence, no response shown by S.P.U.D. subjects would convince a skeptic that fish can feel pain: they could argue, with good evidence, that a fish could show one, or even all, of the responses reviewed here, and yet still be no more sentient than a potato. We discuss the evidence previously used in debates about fish sentience in the section Discussion and Conclusions: Applying Our Approach to the Fish Pain Debate.

But what are S.P.U.D subjects *unable* to do? Can we use this information – as well as current ideas about the correlates and even functions of P-consciousness – to suggest more convincing measures of sentience?

## What *Could* Be Evidence Of P-Consciousness and, More Narrowly, Sentience?

Biologists and psychologists typically assume that P-consciousness is functional, being somehow crucial for flexible, strategic behavior of a greater complexity than the responses of S.P.U.D. subjects [e.g., (25), p. 196, 287–292; (37), p. 186, 189; (33, 65, 116, 134, 143)]. Baars, for instance, proposes nine functions of P-consciousness that include integrating perception, thought and action, adapting to novel circumstances, and providing information to a “self system” [summarized by Blackmore and Troscianko (25), p. 196]. Similarly Ginsburg and Jablonka [e.g., (37), p. 233–237] argue that amongst its hallmarks are: the binding of information about multiple features of the world, and the global accessibility of this information (thanks to long-term memory) for evaluation and use in selective, flexible goal directed behavior. Perhaps these attributes thus capture what's missing in Woolf (71)'s striking contrast between decerebrate and intact rats: the former “react to noxious stimuli with an ‘indifference' such that immediately after application of a noxious stimulus the animals will carry on grooming etc. as if nothing had happened. … no immobility or sustained licking of the injury, and no avoidance of the experimenter”.

Is it possible to be more precise than this, and identify specific, well-operationalized behavioral or cognitive attributes that *require* P-consciousness? The answer is “not quite yet”, which leaves a “theory heavy” or deductive approach currently impossible. But Birch (33) suggests a pragmatic alternative: a “theory light” approach of inference to the best explanation, that “commits to a broad hypothesis about the relation between phenomenal consciousness and cognition… the motivating idea being that phenomenal consciousness does something for cognition”. To build on this constructive proposal, here we suggest four types of candidate measure that seem particularly promising as indicators of sentience. One concerns working memory, and another, forms of operant conditioning: two types of ability that S.P.U.D. subjects seem not to convincingly have. A third type of candidate measure concerns self report. As we saw in the section *Red Herrings:* Responses That Do Not Require Sentience, unaware humans, and also monkeys with blindsight, report sensing nothing (thence having no P-consciousness), even when presented with stimuli that elicit other kinds of response – with the human subjects consistently describing themselves as merely guessing. Finally, higher order cognitive processes such as episodic memory and self-recognition, can yield insights into animal awareness. These have not been studied in S.P.U.D. subjects, and this would likely be impossible, but are arguably reliant on perceptual consciousness. We review each of these below, and also outline how they could be modified to now address questions about sentience: the ethically relevant dimension of P-consciousness at the heart of the fish pain debate. We also suggest how their validity as indicators of sentience could be assessed.

### Tasks Involving Working Memory and Aspects of Operant Conditioning

Working memory tasks require subjects to retain and use information after a delay. The potential role of P-consciousness in these tasks is revealed by some responses that humans with blindsight and similar deficits are unable to make. Above, we reviewed how subjects with blindsight can make visual discriminations, despite their lack of phenomenal sight, including reaching out with the appropriate space between fingers and thumb when asked to grasp objects of different sizes. However, they *cannot* make these size-appropriate adjustments if there is a 2 second delay between the stimulus presentation and the reaching task (95, 144). Likewise, blindsighted subjects successfully reach around obstacles currently presented to their blindfield, but they cannot do this if a 2 second delay is interposed between being presented with the set-up and then performing the task (145). Similar effects of delay have also been reported for the somatosensory equivalent of blindsight, “numbsense”, for both tactile and proprioceptive stimuli [(95) cited by (96)]: subjects can react appropriately only to current inputs, not recent ones whose properties require recall. Furthermore, specific sub-types of trace conditioning, in which the length of the delay is is learned, may also require awareness [see (33)], perhaps because similarly reliant on working memory (146). The role of awareness in human working memory is therefore under intense current investigation, as well as some debate [e.g., (147)]. Specifying particular tasks for animals is thus probably premature at this stage, but this does seem like an exciting research area for those interested in animal consciousness to follow.

One caveat, however: as normally run, working memory tasks typically involve affectively neutral sensory stimuli (e.g., visual cues). These are only relevant for fish and other animals if one is just interested in the general conscious perception of stimuli [cf. (32)]: P-consciousness in its broadest sense. But because of the componential nature of consciousness (see Introduction), such tasks are not useful if one is specifically interested in sentience [i.e., conscious affect, cf. (116)]. Thus, if in the future, certain working memory tasks were robustly validated as requiring the conscious awareness of, say, visual cues, they could then be used to probe whether fish have phenomenal sight: something disputed by those who believe fish have no P-consciousness at all (e.g., Key in prep., pers. comm.). That would be very useful. However, such tasks would *not* address whether fish can experience pain or other conscious affective states. Instead, operant learning might be more suitable for this type of question because of the central roles that valenced stimuli (punishers and rewards) play in the learning of these responses.

Broadly speaking, aspects of operant learning do seem to hold promise as indicators of P-consciousness: for some human operant tasks, both experiment [e.g., (132)] and self-reflection (e.g., recalling or imagining learning such skills as riding a bicycle [(25), p. 94; (37), p. 75]) suggest a role for awareness in their acquisition. As yet, there is also little evidence that S.P.U.D subjects can do this form of learning. This is therefore another highly relevant research topic to keep abreast of. But again, those interested in sentience specifically (not just perceptual aspects of P-consciousness) need to pay careful attention to whether awareness of the operant task, of any discriminative stimuli, and/or of the reinforcer and its affective significance, are being manipulated and assessed in these human studies [cf. e.g., (31, 75)]. Only tasks that manipulate the awareness of reinforcers (or punishers), and find effects, are candidates for identifying sentience, because only these are investigating the role of conscious affect.

Given this, we suggest three possible ways in which operant tasks might fruitfully be investigated for their reliance on sentience. We agree with Paul et al. (116) that “evidence that reinforcement learning in humans requires rewards and punishers to be experienced as conscious feeling states (i.e., of positively or negatively valenced affect) would help shed further light on the potential utility of inferring conscious animal affect from reinforcement”. So, first, one obvious research question is: can humans learn novel operant tasks, from scratch, to gain rewards or avoid punishers of which they are not aware? To investigate this, we thus need to design an experiment to assess whether arbitrary responses can be conditioned *de novo* by subliminal reinforcers. Subjects could perhaps be presented with a variety of manipulanda (e.g., a lever, a push button and a switch). Interacting with one of these yields subliminal reward cues (e.g., imperceptible levels of a rewarding drug, masked smiling faces or piles of money, or incentive cues presented to the blindfields of blindsight subjects); interacting with another yields subliminal punishment cues (e.g., masked angry faces or images of trauma); and interacting with the third yields nothing. Given minimal instruction, and no explicit information on success, would subjects eventually acquire preferences for the positive operant and aversions to the negative operant? A related approach could be to see if a particular novel motor response could be “shaped” (by successive approximation) in naive human subjects by such subliminal reward cues, or perhaps even to see if the other types of S.P.U.D subjects could, despite the challenges involved, likewise be shaped to display novel, arbitrary operants. Failures would be consistent with sentience being necessary for operant learning, as hypothesized, while successes would show it to be unnecessary.

In the latter instance, drilling into *sub-types* of operant learning might then be more useful. Focussing on goal-directed forms of operant learning is one potential strategy. “Conscious awareness of a reward enables individuals to change the strategies they employ to attain that reward”, claim Capa et al. (141). “The possibility remains that some forms of reinforcement-learning, such as goal-directed learning, do indeed require consciously experienced affect to occur“, suggest Paul et al. (116) more modestly. Technically, goal-directed learning is often defined as being sensitive to reinforcer devaluation [e.g., prior satiation of a subject with the particular food they are responding to: reviewed (35); see also (36)]. Now, work by Ziauddeen et al. (138) has already shown that subliminal incentive cues illustrating food winnings of pizza or pie *will* selectively modify the responding of pre-sated subjects. But would such effects of subliminal incentive hold for operants that were conditioned *de novo*, rather than performed to follow instructions? That seems as yet unknown. Other research approaches could be to ask whether subjects will respond flexibly to attain a subliminal reward, for example performing an operant with their mouth if they cannot use a limb, or overcoming obstacles to do so (e.g., moving away items blocking or obstructing the operant apparatus). These are the types of study that would reveal whether goal-directed operant responses do indeed require conscious affect.

Implicit here, especially in those last scenarios, is the idea that an operant response can become motivating and potentially even reinforcing in its own right. And indeed Ginsburg and Jablonka [(37), p. 231–233] propose that operant conditioning involves sentience if it involves second-order conditioning (e.g., the learning of compound sequences by successive chaining, in which each reinforced action becomes a secondary reinforcer that then conditions others). Whether this is correct seems unknown as yet: their idea needs testing. But it is empirically testable. For example, we have seen that decerebrate rats, and spines disconnected from brains, can display Pavlovian conditioning, and also learn responses to avoid shock. But if a neutral tactile cue was repeatedly paired with shock, could they then learn instrumental responses to avoid these CSs? In other words, can a CS become a secondary reinforcer without awareness? If Ginsburg and Jablonka are correct, then the answer would be no. Ginsburg and Jablonka's hypothesis could also readily be tested in humans, in paradigms manipulating awareness of reinforcers and/or awareness of potential CSs: if correct, humans would not be able to chain responses together for subliminal rewards, for example, nor learn operants for subliminal Pavlovian CSs. Such findings, were they to emerge, would be of huge importance, validating new tools for investigating sentience in animals.

### The Self-Report of Sensation

A third deficit in unaware humans is that because of the self-reported lack of relevant sensation, subjects have little to no confidence in the validity of their responses to stimuli that they feel they cannot detect. This can manifest as an unwillingness to wager on choices dependent on those stimuli [even when they make those choices correctly at above-chance levels: e.g., (31, 148), p. 25–26]. So, can we develop tasks that ask animals to self report in ways that are as reliant on P-consciousness as subjective self-report in humans? First, we should clarify what is meant by “self-report”. In human experiments, subjective self reports are elicited responses to requests to introspect, often manifest as freeform verbal descriptions, ticking off subjective states on a written checklist, or marking a Likert Scale. They thus involve arbitrary, learned responses, performed flexibly according to the specific context or task at hand, and their function is conveying information on internal state to an outside observer. Yelping or pulling away a hand from a hot item would thus not be deemed self report, because these are innate, stereotyped responses that would occur even without an audience.

One potential approach to ask animals to self-report how (or even if) they feel, is to see if they can use internal states as discriminative stimuli [(149), Mason et al. in prep]. This is a method widely used in psychoactive drug research, on both humans and animals. Here, subjects are trained to use the presence or absence of a drug-induced state as a cue that guides which of two operants will be reinforced (with food or money, depending on species). Such research reveals some compelling findings, especially for rats. For example, rats' abilities to use a drug as a discriminative stimulus (DS) in lever-pressing tasks are altered by states that in humans would make that drug easier or harder to detect. Thus their use of aspirin as a DS is enhanced if they have potentially painful arthritis (150); while their use of the anxiogenic drug “PTZ” as a DS is abolished by anti-anxiety drugs [e.g., (151)]. Rats also “generalize” between drugs that feel similar to humans, even when the chemical modes of action differ; for instance if trained to use having an alcohol hangover as a DS, rats then choose the hangover lever if subjected to morphine withdrawal (152). Similarly, rats generalize between drug and non-drug treatments that seem likely to have similar subjective effects. For instance, the rats trained to self-report alcohol hangovers also chose the hangover lever if exposed to “jetlag” [an 8 hr time shift: (152)], while rats trained to use PTZ as a DS would pick the PTZ lever if exposed a cat, as if self-reporting similar states of anxiety (153). Such powerful experimental data led Emmet-Oglesby et al. (154) to conclude that this paradigm “provides the most sensitive and accurate behavioral analog in animals to what humans verbally report about subjective drug experiences” [with similar conclusions from other authors in this field; Wood and Lal (155), for instance, argued that this type of animal test is “a bioassay for detecting subjective effects”]. Similar data for fish might therefore be highly persuasive.

But could such results merely reflect a “blindsight-like” guessing: a mere discrimination response that need not reflect underlying awareness? After all, as we have seen for S.P.U.D. subjects, decerebrated pigeons can use colored lights as DSs (128), and humans can use subliminal visual stimuli as DSs [e.g., (121)]. We think several refinements could reduce this risk. One is requiring a performance criterion that exceeds the rather poor discriminative responding shown by these unaware humans and birds [e.g., to require that > 90% responses are cued by the DS+, rather than the 60 or 70% that unaware subjects seem to achieve at best: (121, 128, 132–134), Skora pers.comm.; also Mason et al. in prep.]. To further increase the task's sensitivity to subjects' confidence levels, a second refinement could be to make incorrect guesses costly, and to include an option to “opt-out” [cf. the use of “commentary keys” when studying blindsighted humans [(32), p. 47–48, 230–231] and monkeys (156); see also the opt-out key used in metacognition research by Hampton (157)]. This would essentially ask animals “are you *sure* you sense something?”. Finally, a third option is to mimic some creative recent work on corvid visual perception by Nieder et al. (158), in both imposing a post-stimulus delay before the task and also requiring subjects to select a response from a *choice* of possible actions. This respectively adds an element of working memory (as discussed above), and also requires subjects to use information about their states in a flexible manner [cf. eg., (62)]. We think that Nieder et al. (158)'s (unstated) assumption that their task specifically detected conscious perception is extremely plausible. But importantly, we also think the validity of this assumption is *testable*, for instance by running humans through an identical experiment (with people replacing crows in this visual task) while actually asking them what they can see. Were the resulting data to support Nieder et al.'s assumptions, modified versions of such a task could be powerful tools for accessing animals' subjective states. Thus, overall, refined experiments using internal states as discriminative stimuli in operant tasks could prove very useful for investigating animal sentience.

### Tasks Reliant on Higher Order Consciousness

Turning to cognitive processes seemingly reliant on insight, higher order capacities like self-awareness are generally seen as reflecting P-consciousness (as reviewed in the Introduction). Passing experimental tests for self-reflection or self-awareness is therefore usually taken as evidence of P-consciousness. Below, we discuss two forms of such evidence – mirror self-recognition and episodic memory – and, following the same logic as the previous section, suggest how they might be made more relevant to sentience and thence the fish pain debate.

Mirror self-recognition, as assessed in the famous “mirror mark test”, is one ability of great significance to those interested in P-consciousness. Gallup, who devised this test, sums up what passing it means: “the observer needs to come to the realization that it is their behavior that is the source of the behavior that is being depicted in the mirror” [(159), in a paper that also emphasizes the needs for careful controls in such work]. McFarland [(160), p. 132] also offers a nice analysis of this “kinaesthetic visual matching”: “if an organism has this ability, it looks in a mirror and recognizes that the visual display in the mirror matches its kinaesthetic experience”. In other words, parsing this out into components, to pass this test a subject must have both visual awareness and proprioceptive awareness, as well as the cognitive capacity to spot the contingency between their own movements and their reflection's. Thus, it need not indicate something as grand as a “concept of self” [(25), p. 265], but it still relies on P-consciousness.

So, since cleaner wrasse (*Labroides dimidiatus*) have this ability (161, 162), this is strong evidence of both visual and proprioceptive awareness that, as discussed in the section Tasks Involving Working Memory and Aspects of Operant Conditioning, refutes those who argue that fish have no P-consciousness at all. But this does not yet indicate that the wrasse have a capacity to feel pain or be sentient. To achieve *this*, we therefore need a new type of mirror experiment that is explicitly designed to probe affective states. That is a challenging task, but one approach might be what we call the “mirror test with biting parasite”, in which the mark is not affectively neutral. For example, the subject is exposed to a mirror, to provide opportunities for kinaesthetic visual matching. The subject is then repeatedly exposed to two marks, differing in color or shape, placed on their body in a location they can only see via their reflection (e.g., the head). To add the affective component, one of these (let's say a black triangle; obviously this would be counterbalanced across subjects) is rendered uncomfortable or even painful; the other (let's say a white circle) is not. After several such trials, a mark (now without any associated discomfort) is then placed on the animal's body in a location it *can* see (e.g., its tail, for many fish) (with controls involving presenting the same mark elsewhere, e.g., on an object or the side of the tank). The question would then be, does the subject react differently to the previously nasty black triangle, vs. to the previously benign white circle, when placed on its own body?

Likewise, evidence for episodic memory, the “what, where and when” of past events, is seen as reliant on the flexible use of information about sensations, locations and the passage of time, in a “conscious experience of recollecting” [e.g., (37), p. 440–441], or what Birch et al. (36) call “conscious mental time travel” [see also (33)]. In an elegant experiment on rats, for instance, Ergorul and Eichenbaum (163), taught animals to recall and effectively report on single training episodes, each composed of a series of four odors presented in different places on an open field. The rats were then probed for their abilities to flexibly use different aspects of their experience to solve a new task. Thus, after each training episode, in a probe test differing in format from this episode, rats were offered a choice of two of the four stimuli just presented, chosen at random (either two odor-location pairings, or just two locations, or just two odors), and rewarded for picking the stimulus that had occurred the earliest in the preceding sequence of four. Rats could pass this test. They thus remembered the order of events in unique experiences, and from this flexibly extracted combinations of odor and place information. This, the authors argue, is “consistent with current characterizations of human episodic memory as the capacity to ‘replay' memories as a sequence of events and where they occurred in a previous experience” (although they carefully note this does not prove that rats have the subjective experiences that characterize episodic memory in humans). Again, running humans through identical experiments would be a good way to *test* the intuition that such tasks can only be passed with conscious recall, for instance by seeing if the order of subliminal cues cannot be “replayed” to win a reward (while only that of supraliminal cues can be). But for now, if we assume that this *is* good evidence of P-consciousness of odor, location and the passage of time, then if fish convincingly passed such a test [c.f. (164, 165)], this would reveal that they too have similar capacities. Again, this would defy those who argue that fish do not have the brains for any forms of awareness, but not help those specifically interested in affective, evaluative aspects like pain.

To use episodic memory experiments relevant to sentience specifically, once more we therefore need specifically designed tests that deliberately incorporate an affective component. For example, a “what, where, and *in which state*?” version of Ergorul and Eichenbaum's (163) task might expose animals to locations or sensory cues (e.g., odors) while induced to be in different affective states: perhaps different states of food deprivation (thence potential hunger), different threats of injury (thence potential pain), or differential exposure to rewarding brain stimulation via electrodes or drugs (thence potential pleasure). The probe test could then ask such questions as, can the subjects successfully identify which of two stimuli (locations or odors) had been presented while they were in more negative affective states, versus while they were in more positive states?

## Why Is Sentience Ethically Relevant and What Are The Consequences Of Recognizing It?

As Birch et al. (54) sum up, “if a being is sentient, there are limits on what a human can ethically do to that being”. And currently, fish experience practices that are likely to cause suffering, if they are aware. These include shark-finning, live sushi (“ikezukuri”), and globally, the wild-capture fishing that causes significant bodily injury [e.g., (166, 167)] and affects billions of individual fish (168, 169). So, given uncertainty about fish sentience, what should be done? Some argue that the potential feelings of fish should play no role in their protection, concerned that this could incur costs to industry, research and consumers which – if fish are actually *non-*sentient – would be needless. Thus, Browman et al. (16) worry that “the impact of increasing welfare-related constraints on aquaculture… will leave society less able to produce high-quality protein to feed a still-growing global population”. Indeed this “appeal to consequences” seems to be why, instead of being agnostic, such authors argue that fish are definitely non-sentient [e.g., (6, 16, 170)]. Yet these authors *also* argue that this need not mean that fish are unprotected: that an animal's welfare can be considered separately from whether it is sentient, such that even non-sentient beings should well be cared for. So in this section we ask, is sentience important, or not? And does declaring an organism sentient impose enormous costs on humans?

For many ethicists, sentience *is* crucial for determining whether an animal is entitled to moral consideration of its welfare [e.g., (51–53, 171)]. Varner (172), for example, summarizes a utilitarian ideal of maximizing “aggregate happiness”, from which it follows that moral concern should extend to anything capable of happiness or its opposite, suffering. But even outside utilitarianism, sentience can be held as the key prerequisite for moral concern. For example, DeGrazia [who defines sentience as the ability to have pleasant or unpleasant experiences: e.g., (173)] states: “only beings with interests have moral status, and only sentient beings—who, by definition, have certain mental states—have interests” (53). Consistent with this, much legislation and policy confers special status to sentient organisms. Thus, in Canada, research animals are protected by the CCAC (Canadian Council for Animal Care), but there is no “CCPC” for plants used in research; and for agricultural species there is a National Farm Animal Care Council (NFACC), but no “NFPCC” for crops. Sometimes policy-makers lay out the crucial role of sentience explicitly. The EU, for example, requires its member states to “pay full regard to the welfare requirements of animals” [using a definition of welfare that prioritizes affective states: (174)] since “animals are sentient beings” (175); and Australia's Animal Welfare Strategy states that “sentience is the reason that welfare matters” [also noting that animal welfare reflects the ethical imperative to minimize suffering and consider quality of life: (176)]. In other cases, the role of sentience is instead implied via the use of terms reliant on conscious affective states, such as “distress”, “suffering”, and “humane treatment”. For example in Canada, the CCAC “utilizes affective states as the primary determinant of animal welfare” (177), while internationally, the World Organization for Animal Health (OIE) says that “an animal experiences good welfare if the animal is… not suffering from unpleasant states such as pain, fear and distress” (178). Thus if fish are deemed sentient, this *does matter*: it means they *should* be treated differently from plants, and given extra protection as are mammals and birds.

**Figure 3:**
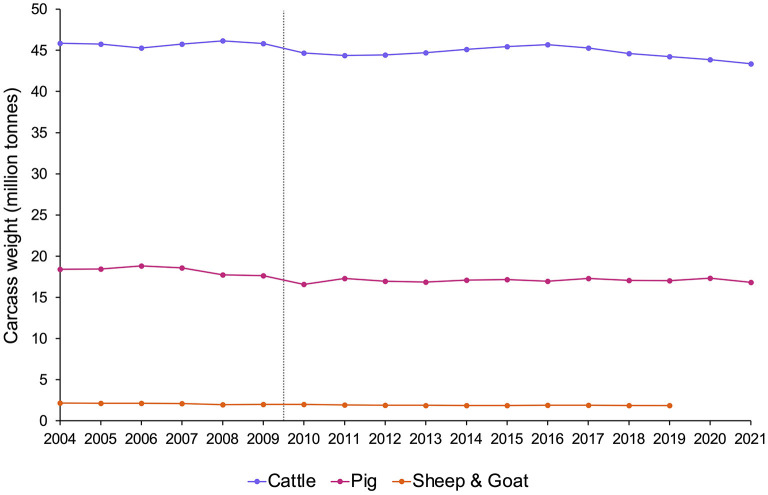
Meat production (million tonnes of carcass weight) in EU countries from 2004–2021 by species, estimated from Eurostat data [sources: (apro_mt_lscatl), (apro_mt_lspig), (apro_mt_lssheep), and (apro_mt_lsgoat)]. The grey dotted line indicates the point at which the Treaty of Lisbon recognized that all animals are sentient (2009). Note that after the Treaty of Lisbon was signed, although animal protections subsequently changed, meat production levels remained the same.

But what if we do not know? “It is sometimes necessary to act on the basis of evidence that does not deliver complete certainty” (54), and when there is scientific doubt about whether the animals covered by these policies are sentient, legislators often implement versions of the “precautionary principle”. This is an approach used to reduce risk when relevant scientific evidence is uncertain or incomplete, especially in cases that may be near a tipping point [e.g., (33, 179)]. For fish, this means balancing the potentially needless cost to industry, research, consumers, etc. that could result from assuming fish *are* sentient when they *are not*, against the risks of significant and potentially unnecessary animal suffering that could result from assuming fish *are not* sentient when they *are*. For many ethicists, policymakers, and welfare scientists (including Victoria Braithwaite, and ourselves) [e.g., (23, 180–182)], the latter's moral weight tips the balance. Will this compromise human interests, as some worry? In an attempt to reassure those concerned about this, Figure 3 presents evidence that recognizing sentience need not impact the numbers of animals used by humans: the Treaty of Lisbon (175) was *not* followed by declining meat production (183), although animal *well-being* is increasingly well-protected by law in EU countries.

## Discussion and Conclusions: Applying Our Approach To The Fish Pain Debate

What *is* it like to be a bass? Some claim that fish have rich experiential lives, able to feel pain, fear, and possibly joy [e.g., (12, 13, 24, 184, 185)]. Others claim that fish are essentially unconscious zombies: that being a bass is like nothing, because fish have no phenomenal experience, not even sight [(6); Key in prep., pers. comm.]. The “fish pain” debate is thus rather polarized. Furthermore, discussion and comment still outweigh new data: only 43% of the citers of Sneddon et al. (9) are experimental papers. This research inertia may stem, at least in part, from the untested assumptions of both “sides”. And of course, it also reflects that the whole broad field of P-consciousness is extremely challenging, rife with complexity, debate, and struggles to tackle what is infamously known as “the hard problem”.

As we have reviewed, P-consciousness is currently impossible to measure, and impossible to assess in non-humans. Indeed even in humans, assessment is imperfect, relying on the veracity and accuracy of self-report. This makes the claims at both these extremes too strong – and the high levels of agnosticism about fish abilities to feel pain, appropriate. Perfect, “bullet-proof” diagnostic markers of sentience simply do not exist, at least as yet. Elwood (186) therefore lamented, “the idea of feelings or consciousness … is impossible to access, and [leads to] arguments that cannot be resolved”. We are sympathetic to this, but somewhat more optimistic. We believe one argument that *can* be resolved is whether sentience matters: it does. Another is whether categorizing fish as sentient will destroy human livelihoods: European data suggest the resulting protections would not necessarily radically hinder animal use (something some may find reassuring, but others, sad). A third argument that we think can be resolved is whether or not to withhold protecting fish until we know they are sentient. The precautionary principle, and the great potential harms done to fish, both indicate that inaction would be ethically worrying; agnosticism about fish sentience is therefore consistent with supporting practical guidelines that choose to protect fish. Indeed, where practices risk extreme pain, we suggest that the guiding question should perhaps not be “is there evidence that this species is sentient?” but instead “are we sure it is not?” Finally, we suggest that empirical research on animal sentience can advance, doing so faster and more constructively, if it is deemed reasonable to treat plants and protozoa, spines disconnected from brains, decerebrate animals and unaware humans (i.e., S.P.U.D. subjects) as not conscious; to treat human self-report as a “gold standard” (as many consciousness researchers do, despite even this being imperfect); and to treat perceptual and evaluative dimensions of P-consciousness as separate and dissociable. We recommend that the questions “*Can S.P.U.D subjects do this?”* and “*In humans, does this always correlate with self-reported feelings, and if so, what type?”* are used to screen all potential indicators of animal P-consciousness. Answering these questions will weed out “red herring” measures that fail to distinguish between the sentient and non-sentient, identify types of indicator that best permit strong inference [e.g., *sensu* (33)], and so assist with both data interpretation and designing new studies.

Applying this approach to current “fish pain” data is revealing: many measures do not survive this screen. For one, S.P.U.D. subjects show diverse unconditioned behavioral responses to noxious stimuli, including avoidance or wound attendance, making it hard to argue that similar responses in fish require awareness or demonstrate true pain rather than mere nociception [cf. e.g., (61, 187), citing (188) on rocking in trout and (189) on tail-beating in zebrafish]. Furthermore, in S.P.U.D. subjects such responses can be modulated, including by analgesics. These means that contrary to several authors [c.f. e.g., (61, 187), citing (188, 190)], the modulation of fish responses to noxious stimuli by analgesics *cannot* be said to indicate pain over nociception; and the same applies to their modulation by food deprivation [c.f. e.g., (61, 187), citing (191)] or conspecific presence [c.f. e.g., (61, 187), citing (192)]. Furthermore, nor does the conditioning of escape responses to locations where a shock was delivered [c.f. e.g., (61), citing (192)], or of approach responses toward locations where nociceptive input is reduced by analgesics [c.f. e.g., (61, 193), reporting unpublished work on zebrafish], demonstrate pain rather than nociception: S.P.U.D subjects are similarly capable of Pavlovian conditioning. This includes trace conditioning in its broadest sense, often erroneously held up as a marker of P-consciousness [c.f. e.g., (194), citing (195)]: this too occurs in S.P.U.D subjects. Braithwaite and colleagues were therefore right to conclude, in one of Victoria's last papers, “trace conditioning is widespread and by itself does not indicate consciousness” (15), a conclusion echoed in this Special Topic collection by Droege et al. (143). And the same also holds for instrumental learning, where pre-existing innate responses change in timing or form to become more effective (e.g., at avoiding punishment). This means that, for instance, shuttlebox learning by fish, where escape responses become directed to particular locations to avoid shock [e.g., (196)], is also not proof of awareness or pain.

Now, authors using such responses to infer pain typically present lists of multiple different responses, to be treated as more convincing if demonstrated *en masse* [e.g., (197) p. 52; (61, 187, 193); see also (33) “theory neutral” approach]. At first this seems reasonable. However, attributes or responses that are as consistent with a lack of awareness as they are with P-consciousness can have little or no value for inferring true pain, regardless of how numerous they are. Of course, some measures might still be useful if their *absence* is revealing; in other words if they are deemed *necessary* for inferring P-consciousness. If a species of fish fails to have nociceptors, for instance [as seems true for at least some elasmobranchs: reviewed by Rose et al. (6), Sneddon (198), Smith and Lewin (199)], then perhaps logically, this is evidence that they cannot feel pain? One challenge would be knowing which these necessary indicators are; and another, distinguishing between true negatives and Type II errors. Nevertheless, thinking more formally about *necessary conditions* for various forms of animal consciousness (as well as the still elusive *sufficient* conditions), and parsing these out clearly, would be useful, not least for making assumptions more explicit than they often are.

In contrast, identifying responses that S.P.U.D subjects seem *unable* to make and ones that in contrast, at least in humans seem to require awareness, highlights other indicators as more useful: better able to permit strong inference (even deduction, if validated using the experiments we suggest). These are measures based on higher order abilities, the self-report of state, working memory, and aspects of operant conditioning. So, what might such indicators reveal about fish?

The apparent intelligence of some fish species [e.g., tool use by tuskfish (200), cooperative hunting by moray eels and groupers (201), numerical competency in angelfish (202), and the mirror self-recognition and episodic memory tasks mentioned above] has sometimes been taken as already sufficient evidence for sentience [e.g., (18)]. For two reasons, we believe this is premature. First, as Massimini and Tononi argue [(45), p. 153], “the mere fact that a behavioral repertoire is complex and “cognitively sophisticated” is not sufficient to clinch the case”. Second, as we have reviewed here, the perceptual and evaluative dimensions of P-consciousness should be considered distinct [following (36)], and these tasks typically provide better evidence of the former than the latter. Nevertheless, the apparent intelligence of such species does bode well for tasks specifically designed to probe sentience, making this an exciting area for future work. Thus, in the section What *Could* Be Evidence of P-Consciousness and, More Narrowly, Sentience?, we suggested modifying existing tests for self-awareness and episodic memory, in order to make them sensitive to sentience (not just the visual, proprioceptive and temporal P-consciousness that they rely on in their current forms). We hope our outlines for a “mirror test with biting parasite”, and for a “what, where, and in which state?” episodic memory task, indicate how such paradigms could be tweaked – both in new assumption-testing experiments on humans, and in new experiments with animals. In that same section, we also suggested ways in which self-report paradigms could be developed for animals, including fish (since when animals are trained to use their internal states as discriminative stimuli, highly revealing insights can emerge, as the drug discrimination literature reveals). We believe there are ways to avoid such tasks' risks of just capturing blindsight-like guessing with no awareness, and again, that the intelligence of some fish bodes well for applying these. Were such affect-sensitive higher-order tasks applied to fish (especially once better validated), we would therefore recommend starting with these species with impressive cognitive abilities.

What about other, seemingly less impressive cognitive abilities? We and others [e.g., (116)] have suggested operant learning as a relevant topic to explore. Our own suggestions about operating learning arise partly from looking for tasks that S.P.U.D. subjects seem unable to do. Of course, absence of evidence is not evidence of absence: the lack of data from S.P.U.D. subjects on the learning of arbitrary operants could reflect a lack of research effort, not a failure of such attempts to work. This therefore identifies another topic for future research, using both S.P.U.D. subjects and humans whose awareness of reinforcers is manipulated: the assessment of whether operant behavior relies on reinforcers producing conscious affective states. (Though note that for such work, we suggest using plants and temporarily unaware humans over highly invasive decerebrate or spinally-transected animals). If this hypothesis was supported, then at least some fish have already shown that they are sentient. These include Siamese fighting fish trained to swim through hoops to display to rivals [e.g., (203)], barramundi (*Lates calcarifer*) trained to touch arbitrary targets (204) and goldfish trained to bump a lever for food (205), as well as all farmed species that use demand feeders to deliver pellets.

More stringently, it could be that not all operant learning requires sentience, but that only some sub-types do. Thus, it could be that Ginsburg and Jablonka's (37) hypothesis about second-order conditioning is correct: again something not yet known, but amenable to empirical test. If their hypothesis is supported, then again there are already some cases demonstrating conditioned responses to secondary reinforcers by fish. In one, for example, Foerder (204) successfully used shaping to train a barramundi to swim 10 feet to touch a secondary reinforcer (a target previously paired with food). In another, an extraordinary experiment demonstrated the abilities of goldfish to learn to jump over a hurdle to modify a CS (lights that must either match or not), in order for this CS to predict no shock rather than shock (206). Thus, if future research does show that second-order conditioning relies on the conscious awareness of reinforcers, then these fish are displaying good evidence for positive affect and pain, respectively. What about the other sub-type of operant learning that we highlighted: goal-directed? It is not yet known whether goal-directed operant responding for food, say, require this reward to induce consciously-experienced positive states, but again this idea is testable. And were the hypothesis supported, then species with flexible forms of foraging, like those using the cooperation and tool use mentioned above, seem ideal for formally investigating whether they can show goal-directed operant behavior, in novel experiments specifically designed to assess this ability.

Operant learning is just one of the many topics tackled in considerable ongoing research by human consciousness researchers (with the use of discriminative stimuli, and working memory being others). We recommend following this fascinating work, for example by attending meetings such those hosted by the *Association for the Scientific Study of Consciousness*: learning about this complex, fast-moving human research is both useful and humbling. Furthermore, increased contact with and interest from those working on animal welfare might encourage such researchers to focus more on understanding sentience (since currently, most of their research efforts focus on perceptual forms of P-consciousness). We hope that our suggestions represent a constructive contribution to the deeply interesting, important, but sometimes frustrating field of animal consciousness. Inspired by Victoria, one of the first to wade into this fray, we look forward to the refinement of these ideas and improved methodologies for probing the puzzle of animal sentience.

## Author Contributions

GM and JL participated in the development and writing of this manuscript. GM developed novel experimental paradigms for probing affective sentience and reviewed ”red herring“ S.P.U.D. subject responses that do not require sentience. JL reviewed the ethical context of sentience and the fish pain literature. All authors contributed to the article and approved the submitted version.

## Conflict of Interest

The authors declare that the research was conducted in the absence of any commercial or financial relationships that could be construed as a potential conflict of interest.

## Publisher's Note

All claims expressed in this article are solely those of the authors and do not necessarily represent those of their affiliated organizations, or those of the publisher, the editors and the reviewers. Any product that may be evaluated in this article, or claim that may be made by its manufacturer, is not guaranteed or endorsed by the publisher.
