# Corrigendum: What Is It Like to Be a Bass? Red Herrings, Fish Pain and the Study of Animal Sentience

**DOI:** 10.3389/fvets.2022.948567

**Published:** 2022-06-07

**Authors:** G. J. Mason, J. M. Lavery

**Affiliations:** Integrative Biology, University of Guelph, Guelph, ON, Canada

**Keywords:** fish, sentience, consciousness, pain, welfare

In the original article, there was a miswording. This special edition focuses on the legacy of Dr. Victoria Braithwaite, but the wording of the second sentence in our abstract “*Thanks to Braithwaite's discovery of trout nociceptors, and concerns that current practices could compromise welfare in countless fish, this issue's importance is beyond dispute”* accidentally implied this discovery was her achievement only, when of course it involved others too. To better sum up Dr. Braithwaite's impact, we have now replaced “*discovery of trout nociceptors”* with “*research leadership”* in the second line of the abstract. This does not change the scientific conclusions of the article in any way. The original article has been updated.

## Publisher's Note

All claims expressed in this article are solely those of the authors and do not necessarily represent those of their affiliated organizations, or those of the publisher, the editors and the reviewers. Any product that may be evaluated in this article, or claim that may be made by its manufacturer, is not guaranteed or endorsed by the publisher.

